# Akt Inhibition Promotes ABCA1-Mediated Cholesterol Efflux to ApoA-I through Suppressing mTORC1

**DOI:** 10.1371/journal.pone.0113789

**Published:** 2014-11-21

**Authors:** Fumin Dong, Zhongcheng Mo, Walaa Eid, Kevin C. Courtney, Xiaohui Zha

**Affiliations:** 1 Chronic Disease Program, Ottawa Hospital Research Institute, Ottawa, Ontario, Canada; 2 Department of Biochemistry, Microbiology & Immunology, University of Ottawa, Ontario, Canada; Simon Fraser University, Canada

## Abstract

ATP-binding cassette transporter A1 (ABCA1) plays an essential role in mediating cholesterol efflux to apolipoprotein A-I (apoA-I), a major housekeeping mechanism for cellular cholesterol homeostasis. After initial engagement with ABCA1, apoA-I directly interacts with the plasma membrane to acquire cholesterol. This apoA-I lipidation process is also known to require cellular signaling processes, presumably to support cholesterol trafficking to the plasma membrane. We report here that one of major signaling pathways in mammalian cells, Akt, is also involved. In several cell models that express ABCA1 including macrophages, pancreatic beta cells and hepatocytes, inhibition of Akt increases cholesterol efflux to apoA-I. Importantly, Akt inhibition has little effect on cells expressing non-functional mutant of ABCA1, implicating a specific role of Akt in ABCA1 function. Furthermore, we provide evidence that mTORC1, a major downstream target of Akt, is also a negative regulator of cholesterol efflux. In cells where mTORC1 is constitutively activated due to tuberous sclerosis complex 2 deletion, cholesterol efflux to apoA-I is no longer sensitive to Akt activity. This suggests that Akt suppresses cholesterol efflux through mTORC1 activation. Indeed, inhibition of mTORC1 by rapamycin or Torin-1 promotes cholesterol efflux. On the other hand, autophagy, one of the major pathways of cholesterol trafficking, is increased upon Akt inhibition. Furthermore, Akt inhibition disrupts lipid rafts, which is known to promote cholesterol efflux to apoA-I. We therefore conclude that Akt, through its downstream targets, mTORC1 and hence autophagy, negatively regulates cholesterol efflux to apoA-I.

## Introduction

Epidemiological studies have long established an inverse association between plasma HDL levels and coronary heart disease (CHD). However, simply raising total plasma HDL level has not been successful in preventing CHD [1], [2]. This is thought to be due to substantial heterogeneity in plasma HDL. Only one sub-type, namely lipid poor pre-β HDL, is capable of efficiently removing excess cholesterol from the peripheral tissues including coronary arteries and thus offers protection for CHD. Indeed, a recent landmark study convincingly showed that the capacity of the plasma to remove cholesterol from cultured macrophages, which functions as a readout of plasma pre-β HDL contents, is the most effective indicator for CHD risks [3].

Pre-β HDL depends on ATP-binding cassette transporter A1 (ABCA1) to remove cholesterol. Identification of genetic defects causing Tangier disease has revealed a key role of ABCA1 [4]–[7]. Mutated ABCA1 fails to mediate cholesterol efflux to lipid poor pre-β HDL or its major protein apoA-I. Consequently, Tangier patients present much increased risk of CHD [8]. Thus, enhancing ABCA1 function is likely a highly effective strategy to prevent CHD, parallel to raising pre-β HDL levels. However, the precise molecular mechanisms by which ABCA1 mediates cholesterol efflux to apoA-I remains elusive, which presents a major barrier for the development of pharmacological interventions.

Recent studies have implicated many signaling proteins in ABCA1 function and apoA-I lipidation [9]. For example, greater than ten kinase pathways have already been proposed to modulate ABCA1 activity, including PKA, PKC, Cdc42, CK2 and JAK2 [10]–[17]. We reported earlier that apoA-I acquires cholesterol primarily at the plasma membrane [18]. However, most excess cellular cholesterol in mammalian cells is stored as cholesterol ester (CE) in lipid droplets, such as those found in foam cells. One of the rate-limiting steps for cholesterol removal by ABCA1 is CE hydrolysis that supplies cholesterol to the plasma membrane [19]. Efficient CE hydrolysis promotes ABCA1-mediated cholesterol efflux and reverses foam cell formation.

Recently, cholesterol efflux from macrophages was shown to require autophagy, a catabolic process that engulfs lipid droplets and delivers CE to the lysosomes for hydrolysis [20]. Interestingly, autophagy is suppressed by mTORC1 [21], a master nutrient sensor that is hyper-activated in both diet-induced and genetically obese animals [22]. In addition to nutrients (i.e. glucose and amino acids), mTORC1 is activated by growth factors through Akt [23]. Akt phosphorylates tuberous sclerosis complex (TSC), an endogenous mTORC1 inhibitor, to disable TSC [24], [25]. This leads to mTORC1 activation and autophagy suppression. Akt is therefore likely a negative-regulator of autophagy and potentially a negative regulator of ABCA1-mediated cholesterol efflux to apoA-I.

In this report, we tested the effect of Akt inhibition on ABCA1-mediated cholesterol efflux to apoA-I. Our results demonstrated for the first time that Akt negatively influences cholesterol efflux to apoA-I, most likely through its impact on TSC. We also show that Akt inhibition enhances autophagy and increases plasma membrane cholesterol accessibility to extracellular acceptors. We speculate that both processes contribute to more efficient cholesterol efflux to apoA-I.

## Methods

### Materials and reagents

Cell culture growth medium, antibiotics (penicillin/streptomycin, or P/S) and fetal bovine serum (FBS) were purchased from Invitrogen (Burlington, ON). Rabbit polyclonal anti-ABCA1 antibody was from Novus Biological Inc. (Littleton, CO). Rabbit polyclonal anti-Akt, -pAkt (ser473), -LC3, -flotillin-2 antibodies were from Cell Signaling. Rabbit monoclonal anti-pS6K was purchased from Millipore and rabbit polyclonal anti-S6K was from Santa Cruz. Anti-mouse HSP-70 monoclonal antibody was from BD Biosciences (Mississauga, ON) and peroxidase-conjugated sheep anti-mouse and donkey anti-rabbit IgGs from Jackson Immunoresearch Laboratories (West Grove, PA). [^3^H] cholesterol was purchased from PerkinElmer-Canada Inc. (Vaudreuil-Dorion, QC). Purified human apoA-I was from Biodesign International (Saco, ME). DEBC (10-[4'-(*N*,*N*-Diethylamino)butyl]-2-chlorophenoxazine hydrochloride) and Torin 1 were from Tocris Bioscience. Ly294002, Akt1/2 and rapamycin were from Sigma. Mifepristone, T0901317, methyl-β-cyclodextrin (MCD), bovine serum albumin (BSA) were from Sigma. Scintillation liquid ScintiSafe Gel Cocktail was from Fisher (Whitby, ON).

### Cell culture

Baby hamster kidney (BHK) cell lines were generous gifts from Drs. Oram and Vaughan (University of Washington, Seattle, WA) [26] and RAW 264.7 macrophages (RAW), an adherent mouse macrophage cell line, was purchased from ATCC (Manassas, VA). BHK cells stably expressing an mifepristone-inducible vector with human ABCA1 (ABCA1) or mutant A937V ABCA1 (A937V) gene insert were prepared as described previously [26]. RAW cells endogenously express ABCA1 upon induction with an LXR-agonist, T0901317. Min6, a pancreatic beta cell line, was from Dr. Screaton (Children's Hospital of Eastern Ontario, Ottawa) [27], whereas HepG2, a hepatic cell line, was from Dr. Lagace (University of Ottawa)[28]. Mouse embryonic fibroblasts (MEF), both wt and TSC2^-/-^, were generously provided by Dr. Guan (University of California, San Diego) [29].

All cell lines were maintained in Dulbecco's modified medium (DMEM) supplemented with 10% FBS and 1% P/S (100 units/mL penicillin and 100 µg/mL streptomycin) at 37°C in a 5% CO_2_ incubator. To induce ABCA1 expression, BHK cells were incubated for 18–20 hours with 10 nM mifepristone in DMEM plus 1 mg/ml BSA. Similarly, RAW, HepG2, Min 6 and MEFs were incubated 18–20 hours in DMEM/BSA medium plus 10 µM T0901317. In most of the experiments, Mock or mutant BHK cells and non-induced cells were used as negative controls.

### Cholesterol efflux

Cells were seeded in 24-well plates and incubated with growth medium containing 1 µCi/ml [^3^H]-cholesterol for 24 hours to label cells to equilibrium. The medium was then replaced by fresh DMEM containing 1 mg/ml BSA plus mifepristone for BHK cells or T0901317 for other cell types as described above. For cholesterol efflux, cells were washed and incubated for 2 hours at 37°C either with apoA-I (5 µg/ml) in DMEM/BSA or without. At the end of the efflux period, the medium was collected and centrifuged at 500×*g* to remove detached cells and cell debris. The cell-free supernatant was then combined with scintillation liquid. The amount of [^3^H]-cholesterol in the medium was determined with scintillation counter (Beckman LS 6500) as counts per minute (cpm). The remaining adherent cells were lysed in 1 N NaOH overnight and the cpm of the lysate was measured. Efflux was expressed as the percent [^3^H]-cholesterol in the medium over total [^3^H]-cholesterol cholesterol (medium plus cell-associated).

### Immunoblotting

Cells were placed on ice, washed twice with cold PBS and then lysed with SDS buffer (50 mM Tris-Cl pH 6.8, 100 mM dithiothreitol, 2% SDS, 10% glycerol, and 1 tablet each protease and phosphatase inhibitor per 10 mL buffer). A volume of 100 µL lysis buffer was used in each of 60 mm dishes. Lysates were sonicated for 10–15 seconds to shear DNA and then heated to 75°C for 5 min. Lysates were then centrifuged for 1 min at 13,000 g and protein contents in the supernatant were quantified. For electrophoresis, samples were prepared by mixing 4× SDS loading buffer (200 mM Tris HCl, pH 6.8, 400 mM dithiothreitol, 8% SDS, 40% glycerol, 0.4% bromophenol blue) with 15–25 µg of cell lysates. Samples were loaded onto 10% acrylamide resolving gels (10% acrylamide mix (9.67% acrylamide, 0.33% bis). Separated proteins were transferred to PVDF membranes. PVDF membranes were blocked with TBS-T (50 mM Tris HCl, pH 7.4, 150 mM NaCl, 0.1% Tween-20) containing 2.5% BSA for 30 min and then incubated with primary antibody with gentle agitation overnight at 4°C. After 3 washes with TBS-T (5 min each), membrane was incubated and gently agitated for 2 hours in secondary antibody in TBS-T with 1% BSA. PVDF membranes were then incubated for 1 minute with 1 mL chemiluminescent substrate and developed with x-ray film.

### Lipid analysis for free cholesterol and cholesterol ester

BHK cells were seeded in 6-well plates. After incubated with 1 mg/ml BSA/DMEM plus 10 nM mifepristone for 18 hours, cells were treated with 50 µM DEBC for 2 hours. Then 1000 µl isopropanol was added to each well, rocked at room temperature for 10 minutes and put at 4°C for overnight. Collect the isopropanol to glass tubes and dry down under N_2_ for 90 minutes. The dried samples were diluted by 500 µl 1× Reaction Buffer from an Amplex Red Cholesterol Assay Kit, Invitrogen. A volume of 50 µl was used for each reaction. Meanwhile, 1000 µl NaOH (1 M) were added to each wells for solubilizing cells and measuring the protein concentration after removed the isopropanol. Total cholesterol (TC) and free cholesterol (FC) was measured by a commercial cholesterol assay kit (Amplex Red Cholesterol Assay Kit, Invitrogen) according to the manufacturer's instruction.

### Detergent-free sucrose gradient floatation

The analysis was carried out following an established protocol [30]. Briefly, BHK cells from 10-cm dish after PBS-wash were scraped into 500 ul buffer A (500 mM sodium carbonate, 150 mM NaCl, pH 11). Homogenization was carried out by a sonicator (two 10-s bursts). The homogenate with 1.5 mg of proteins in 2 ml buffer A were mixed with 2 ml buffer B (80% sucrose, 25 mM MES, 150 mM NaCl, pH 6.5), then placed at the bottom of an ultracentrifuge tube. A 5–40% discontinuous sucrose gradient was overlaid above (4 ml of 5% sucrose/4 ml of 40% sucrose; both in a buffer containing 250 mM sodium carbonate, 25 mM MES, 150 mM NaCl, pH 6.5). The samples were centrifuged at 39000 rpm at 4°C for 20 h. 12 fractions of 1 ml each from the top to bottom of the tube were collected.

For probing membrane protein markers in the fractions [31], 0.8 ml of methanol were added to each 0.2 ml of sucrose gradient fractions in an eppendorf tube. Samples were vortexed and centrifuged at 9000 g for 10 sec. 0.2 ml of chloroform were added to each sample followed by vortex and centrifugation for 10 sec. After adding 0.6 ml of water, samples were vortexed and centrifuged at 13000 rpm for 1 min. The upper phase was removed and discarded. 0.6 ml of methanol was added to the rest of lower phase and interphase. The samples were mixed and centrifuged at 13000 rpm for 2 min. The supernatant was removed and the protein pellets were dried under speedvac. Proteins were then solubilised in SDS buffer for SDS-PAGE analysis.

### Statistical Analyses

Statistical comparisons between groups were performed with PRISM software (GraphPad InStat v3.05). All other data are presented as mean ± standard deviation (SD). The statistical significance of differences between groups was analyzed by Student's *t* -test. Differences were considered significant at a *P* value <0.05.

## Results

**Figure 1:**
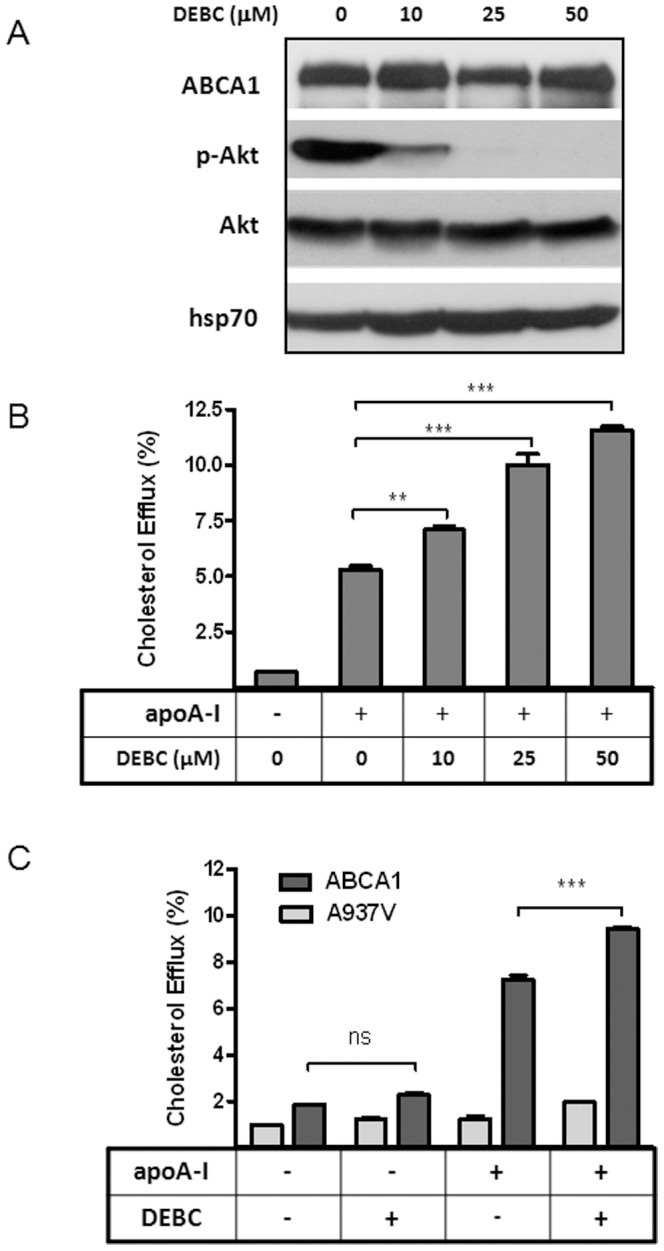
Akt inhibition by DEBC enhances cholesterol efflux to apoA-I specifically from ABCA1-expressing BHK cells. ***A*** *)* BHK-ABCA1 cells were induced with mifepristone (10 nM) overnight and then incubated with indicated doses of DEBC for 2 h. Cells were then lysed and Western-blotted for ABCA1, phosphorylated Akt (p-Akt) and total Akt. Hsp70 was also blotted as loading control. ***B*** *)* BHK-ABCA1 cells were labeled with [^3^H] cholesterol and induced overnight as above. After 2 h incubation with BSA (1 mg/ml) or BSA plus apoA-I (5 µg/ml), cholesterol efflux was measured as described in the Methods section. Some of the cells were also incubated with indicated doses of DEBC, in addition to apoA-I, during 2 h efflux period. ***C*** *)* BHK-ABCA1 and BHK-A937V cells were induced with mifepristone (10 nM) overnight. 2 h Cholesterol efflux was measured as above either with or without DEBC (25 µM). Results are presented as the average of triplicate wells with standard deviation and representative of more than three independent experiments. *** P<0.0001 and **P<0.001 vs apoA-I only.

**Figure 2:**
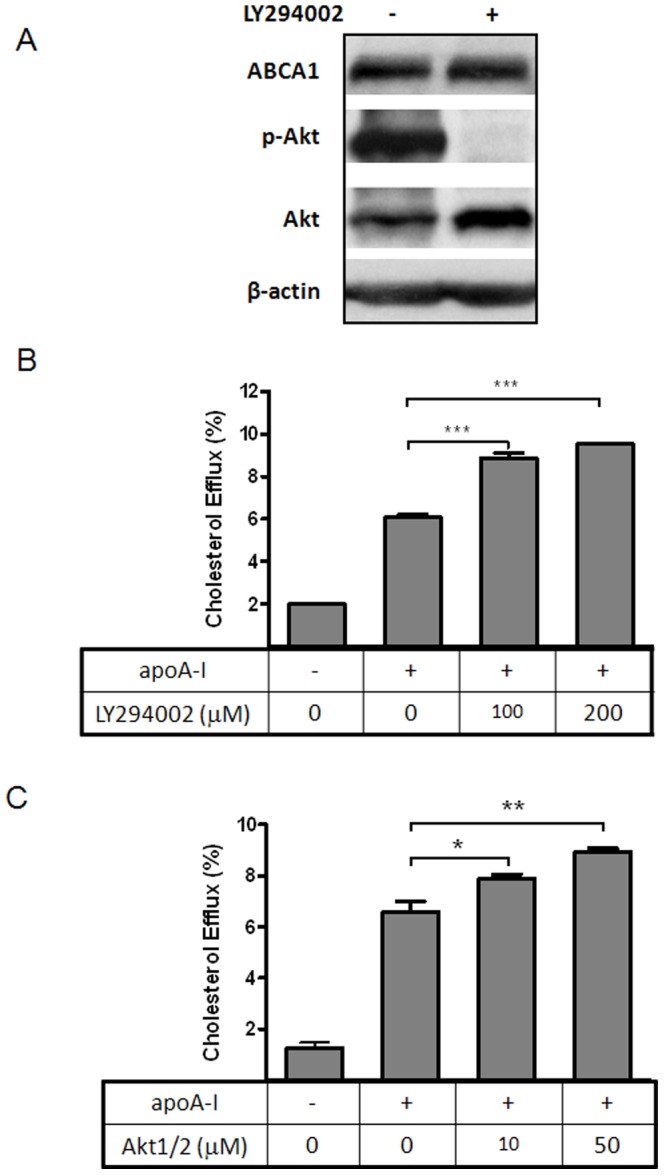
Akt inhibition by LY294002 or Akt1/2 also enhances cholesterol efflux to apoA-I from ABCA1-expressing BHK cells. ***A*** *)* BHK cells were induced as in Fig. 1 and then incubated with LY294002 (200 µM) for 2 h. Cells were then lysed and analysed for ABCA1, phosphorylated Akt (p-Akt) and total Akt by Western-blotting. Hsp70 was also blotted as loading control. ***B & C*** *)* BHK cells were labeled with [^3^H] cholesterol and induced with 10 nM mifepristone overnight. Cholesterol efflux was measured after 2 h incubation with BSA (1 mg/ml) or BSA plus apoA-I (5 µg/ml). Some of cells were also incubated with indicated doses of LY294002 (***B***) or Akt1/2 (***C***), in addition to apoA-I, during 2 h efflux period. Results are presented as the average of triplicate wells with standard deviation and representative of more than three independent experiments. *** P<0.0001, **P<0.001 and *P<0.05 vs apoA-I only.

In searching for intracellular targets that enhance ABCA1 function, we tested various inhibitors using BHK cells that inducibly express wt and mutant ABCA1, a well-established cell model for ABCA1 study [26]. We found that Akt inhibition specifically increases cholesterol efflux to apoA-I in an ABCA1-dependent manner. As shown in **Fig. 1**, Akt inhibitor DEBC dose-dependently inhibits Akt phosphorylation without changing ABCA1 expression (**Fig. 1 A**). DEBC also leads to a dose-dependent increase of cholesterol efflux to apoA-I (**Fig. 1 B**). Significantly, DEBC is only able to enhance cholesterol efflux from cells expressing fully functional ABCA1. It has little effect on BHK cells with no ABCA1 expression (data not shown) or BHK cells expressing a dysfunctional mutant form of ABCA1 (A937V) (**Fig. 1 C**). Similarly, LY294002, an inhibitor of PI3 kinase upstream of Akt, potently inhibits Akt phosphorylation and enhances cholesterol efflux (**Fig. 2 A & B**). Furthermore, another structurally unrelated Akt inhibitor, Akt1/2, is also able to promote cholesterol efflux (**Fig. 2 C**). Thus, inhibition of Akt promotes cholesterol efflux and this action is specific to ABCA1.

**Figure 3:**
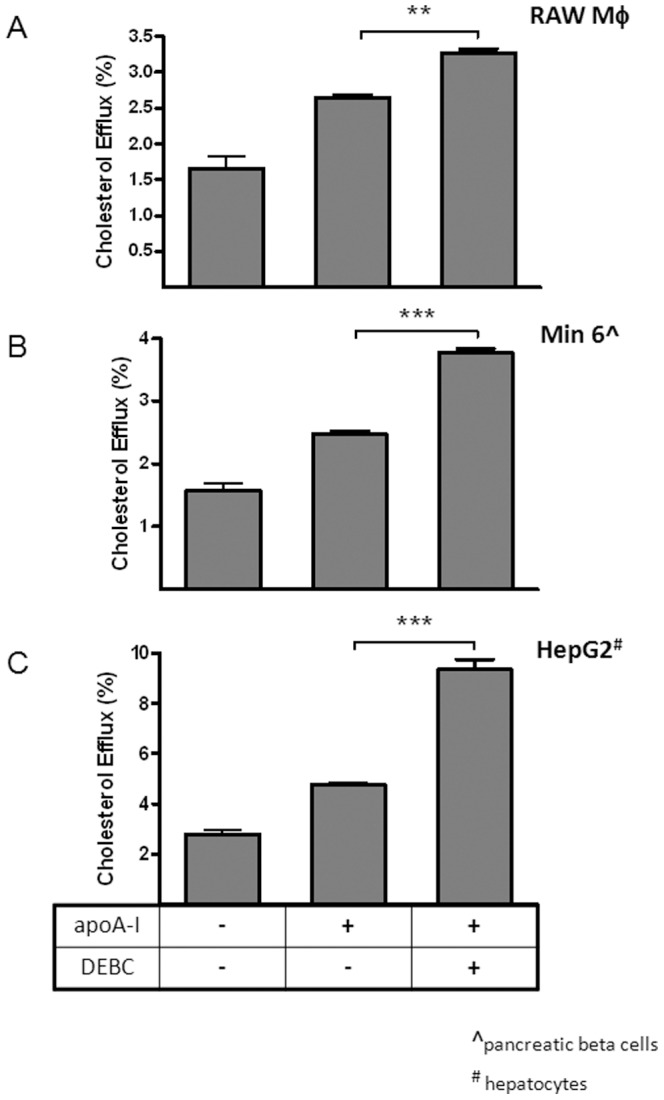
Akt inhibition by DEBC enhances cholesterol efflux to apoA-I from macrophages, pancreatic β cells and hepatocytes. Raw macrophages, Min6 and HepG2 cells were labeled with [^3^H] cholesterol for 1 day and then incubated with T0901317 (10 µM) overnight. Cholesterol efflux was then performed with or without apoA-I (10 µg/ml) for 2 h. Some of the cells were also incubated with DEBC (50 µM), in addition to apoA-I, during 2 h efflux period. Results are presented as the average of triplicate wells with standard deviation and representative of more than three independent experiments. *** P<0.0001 and **P<0.001 vs apoA-I only.

We then tested whether the Akt effect is applicable to other cell types known to express endogenous ABCA1. As shown in **Fig. 3**, DEBC is able to boost cholesterol efflux to apoA-I from RAW (macrophages), Min 6 (pancreatic beta cells) and HepG2 (hepatocytes), which suggests a rather general impact of Akt on cholesterol efflux process, not at all limiting to ABCA1-expressing BHK cells.

**Figure 4:**
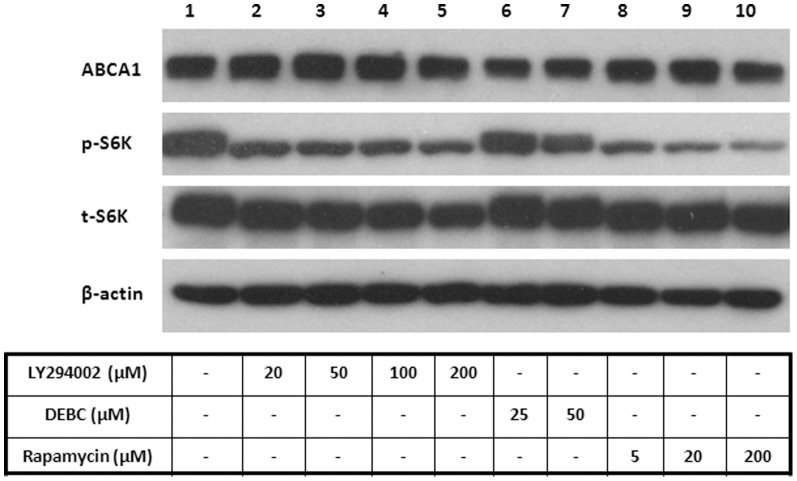
Akt inhibition suppresses mTORC1 activity. BHK-ABCA1 cells were induced overnight with mifepristone (10 nM) and then incubated with DEBC, LY294002 or rapamycin at indicated concentrations for 2 h. Cells were then lysed and analyzed for ABCA1 and phosphorylated S6K by Western blotting. Actin was also blotted as loading control.

We next searched for downstream targets of Akt that might be involved in regulating cholesterol efflux. One of the potential targets is mTORC1, a key regulator of wide range of metabolic functions including lipogenesis [22]. We first investigated whether Akt inhibitors influence mTORC1 activity. As shown in **Fig 4**, both LY294002 and DEBC suppress mTORC1 activity, evidenced by decreased phosphorylation of S6K, an mTORC1 target (lane 2–8). An mTORC1 inhibitor, rapamycin, also suppresses S6K phosphorylation (lane 9–11), as expected. Curiously, although LY294002 potently blocked S6K phosphorylation at low concentrations, such as 20 and 50 µM, cholesterol efflux to apoA-I was not affected at these concentrations (data not shown).

**Figure 5:**
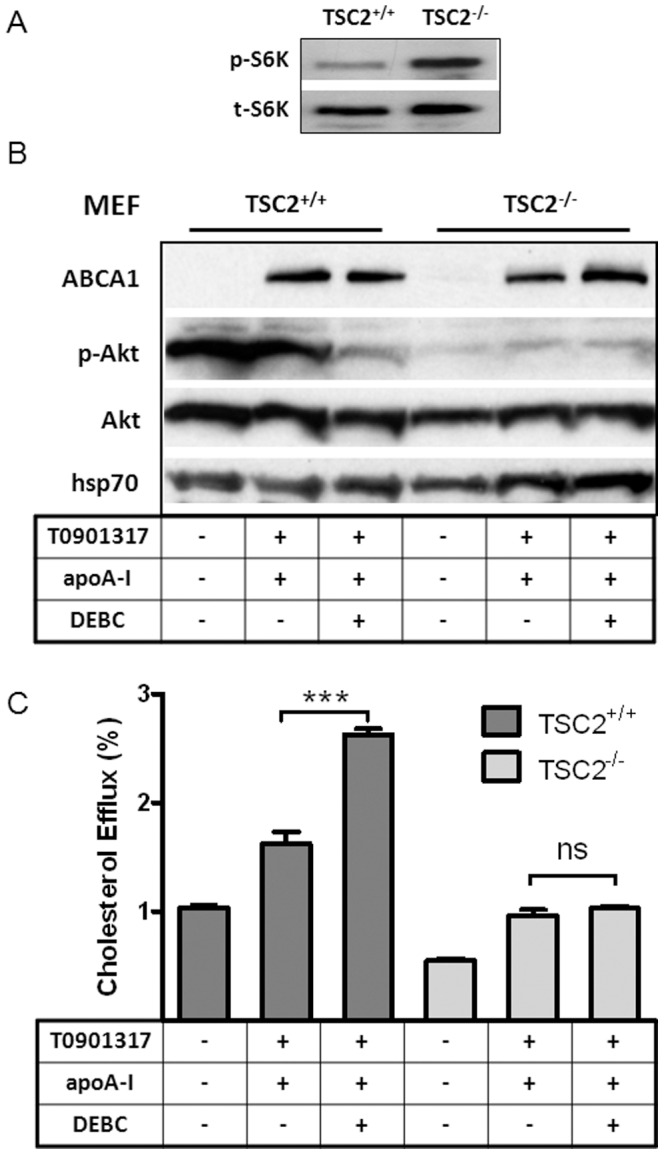
Akt inhibition enhances cholesterol efflux to apoA-I from wt MEFs, but not from TSC2^-/-^ MEFs where mTORC1 is constitutively activated. ***A*** *)* Mouse embryonic fibroblasts (MEFs), wt and TSC2^-/-^, were induced overnight with T0901317 (10 µM). Cell lysates were collected for Western blotting for phosphorylated S6K and total S6K. ***B*** *)* wt and TSC2^-/-^ MEFs were incubated with or without T0901317 (10 µM) overnight. Some of cells were then incubated with apoA-I (10 µg/ml) or apoA-I plus DEBC (25 µM) for 2 h. The expression of ABCA1, phosphorylated Akt, total Akt and loading control hsp70 were analyzed by Western blotting. ***C*** *)* wt and TSC2^-/-^ were labeled with [^3^H] cholesterol for 1 day and then incubated with or without T0901317 (10 µM) overnight. Some of cells were then incubated with apoA-I (10 µg/ml) or apoA-I plus DEBC (25 µM) for 2 h to analyze cholesterol efflux. Data presented as the average of triplicate wells with standard deviation and representative of at least three independent experiments. *** P<0.0001 vs apoA-I only.

We next asked whether Akt requires mTORC1 to exert its negative regulation on cholesterol efflux to apoA-I. mTORC1 activity is suppressed by tuberous sclerosis proteins 1 and 2 complex (TSC1/2). Activated Akt phosphorylates TSC1/2 to release this suppression, whereby activating mTORC1 [24], [25]. Consequently, deletion of TSC1 or TSC2 (both are required for complex formation) leads to constitutive mTORC1 activation, regardless of upstream signals [25]. Consistent with this notion, mTORC1 activity in TSC2^-/-^ MEFs is persistently elevated, shown by high p-S6K levels (**Fig. 5 A**). Similar to its effect in other cell types, DEBC blocks Akt activation and increases cholesterol efflux to apoA-I in wt MEFs (**Fig. 5 C**, left three columns). In TSC2^-/-^ MEFs, Akt activity is low (**Fig. 5 B**, right columns), due to a feedback regulatory mechanism [32]. Still, TSC2^-/-^ MEFs are less efficient in cholesterol efflux to apoA-I, regardless of the lower basal Akt activity. Importantly, DEBC is no longer able to enhance cholesterol efflux to apoA-I in TSC2^-/-^ MEFs. ABCA1 is similarly expressed in TSC2^-/-^ MEFs as in wt MEFs. This supports the notion that mTORC1 is downstream of Akt in suppressing cholesterol efflux to apoA-I.

**Figure 6:**
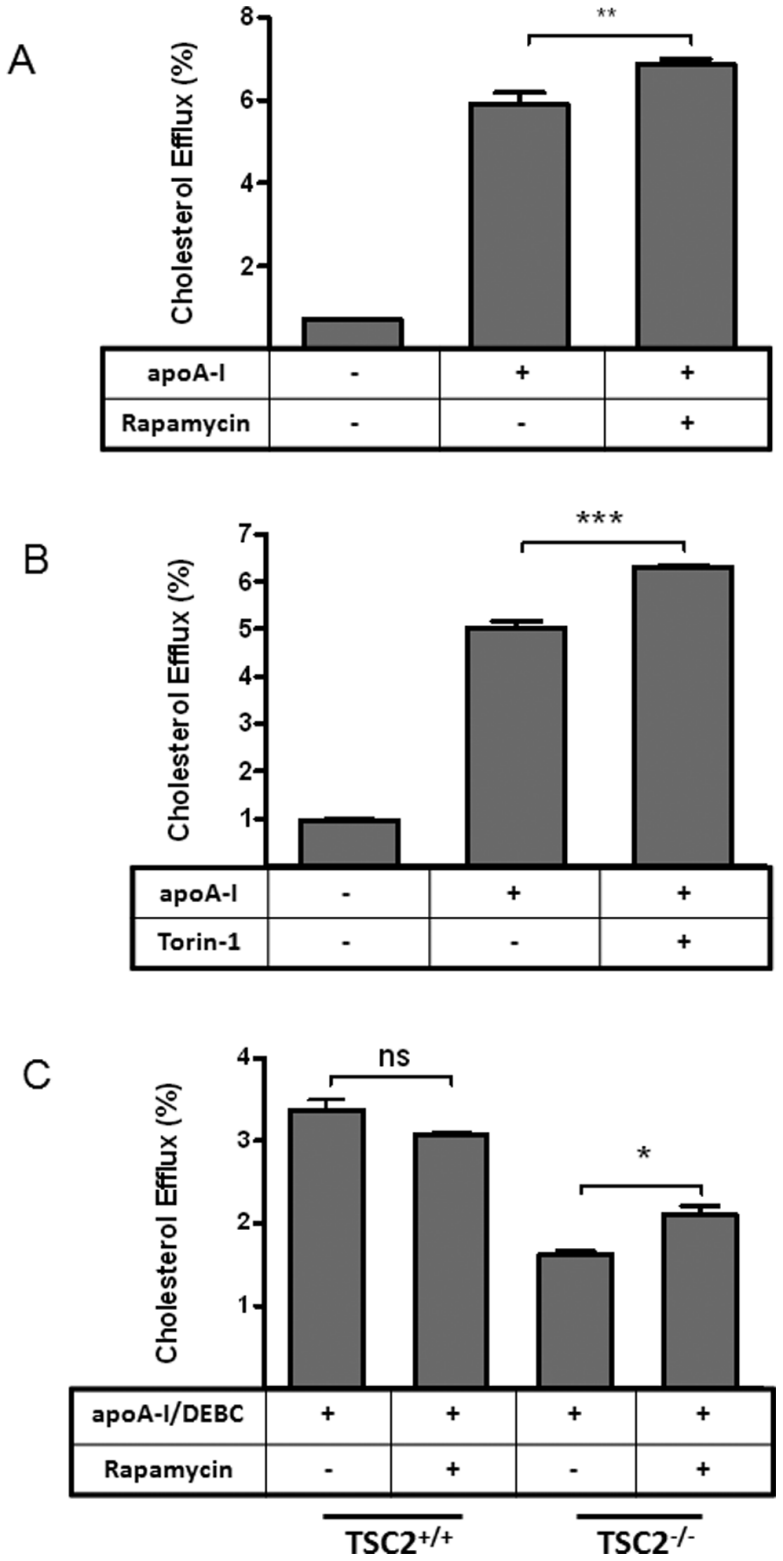
mTORC1 inhibition enhances cholesterol efflux to apoA-I from ABCA1-expressing BHK cells and TSC2^-/-^ MEFs could be partially rescued by mTORC1 inhibition. ***A&B*** *)* BHK-ABCA1 cells were labeled with [^3^H] cholesterol for one day and induced with mifepristone (10 nM) overnight. Cholesterol efflux was measured as the percentage of [^3^H] cholesterol in the medium after 2 h incubation with apoA-I (5 µg/ml) with or without (A) rapamycin (20 nM) or (B) Torin-1 (250 nM). ***C*** *)* MEFs, wt and TSC2-/-, were labeled with [^3^H] cholesterol for 1 day and then incubated with or without T0901317 (10 µM) overnight. Cells were then incubated with apoA-I (10 µg/ml), DEBC (25 µM) and with or without rapamycin (20 nM) for 2 h to analyze cholesterol efflux. Data presented as the average of triplicate wells with standard deviation and representative of at least three independent experiments. *** P<0.0001, **P<0.001 and *P<0.05.

To further confirm the involvement of mTORC1, we next tested the effect of mTORC1 inhibitors. Two commonly used mTORC1 inhibitors, rapamycin and Torin-1, are able to enhance cholesterol efflux to apoA-I in ABCA1-expressing BHK cells (**Fig. 6 A & B**). Additionally, rapamycin has limited effect on DEBC treated wt MEFs, presumably because mTORC1 is already suppressed. However, in TSC^-/-^ MEFs, rapamycin significantly enhances cholesterol efflux (**Fig. 6 C**), though it is still short of full recovery. Both rapamycin and Torin-1 are known to only partially block mTORC1 functions [33], which could explain the partial recovery of cholesterol efflux by rapamycin above. Nevertheless, together with the results from TSC2^-/-^ MEFs, this set of experiments further supports mTORC1 as the downstream effector of Akt, through which Akt suppresses cholesterol efflux to apoA-I.

**Figure 7:**
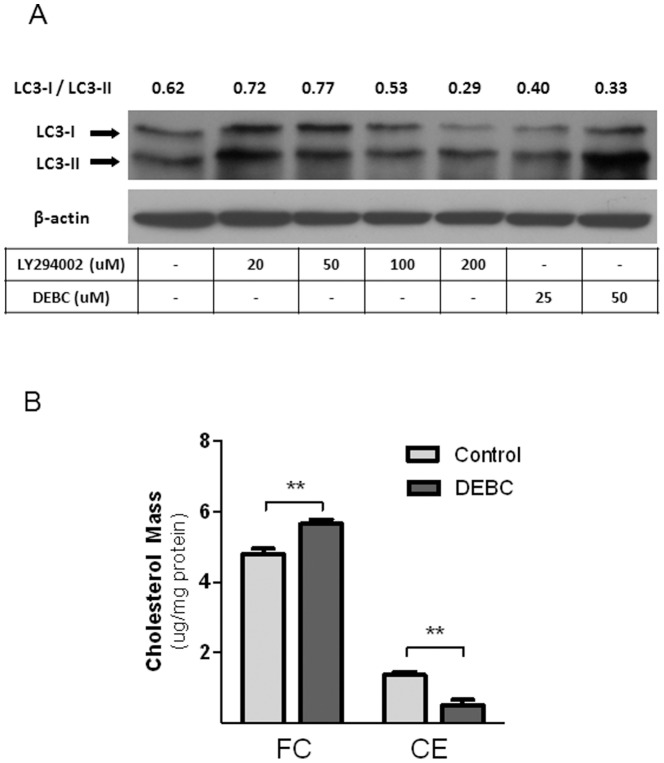
Akt inhibition by DEBC promotes autophagy and decreases cellular CE contents. ***A*** *)* BHK-ABCA1 cells were induced overnight with mifepristone (10 nM) and then incubated with DEBC or LY294002 at indicated concentrations for 2 h. Cells were then lysed and analyzed for LC3 by Western blotting. Actin was also blotted as loading control. The ratio of LC3-I/LC3-II is presented in the top row. ***B*** *)* BHK-ABCA1 cells were treated with DEBC or without for 2 h before lipid extraction and cholesterol mass analysis. Data represents the average of three independent experiments with standard deviation. **P<0.01.

As mentioned earlier, activated mTORC1 is known to suppress autophagy [21]. Autophagy is recently reported to promote intracellular cholesterol trafficking in macrophages, thereby facilitating ABCA1-mediated cholesterol efflux to apoA-I [20]. Interestingly, PI3K also suppresses autophagy, although it could also promote autophagy under various circumstances [34]. We therefore tested whether Akt inhibition by LY294002 or DEBC stimulated autophagy. Autophagic function is commonly detected by the lipidation of LC3, which converts LC3-I to LC3-II [35]. More LC3-II relative to LC3-I (hence lower the ratio of LC3-I/LC3-II) indicates higher autophagy activity. Judging from the ratio of LC3-I over LC3-II, DEBC consistently enhanced autophagy (**Fig. 7 A**). LY294002 also accelerated autophagy, but only at high concentrations (100 and 200 µM). This is in agreement with our observation that LY294002 only promotes cholesterol efflux at higher dosages (Fig. 2). In addition, increased autophagy should facilitate hydrolysis of cholesterol ester (CE). We indeed observed significant decrease of CE mass with correspondent increase in free cholesterol (FC) contents in cells treated with DEBC for 2 h, in comparison with control cells (**Fig. 7 B**). Noticeably, this decrease occurs in the absence of apoA-I. Hence, it could not be due to net loss of cholesterol through efflux from cells (see Fig. 1 C). It is thus likely that cellular free cholesterol (FC) pool is enlarged by Akt inhibition. An enlarged FC pool could in theory lead to more cholesterol supplied to the plasma membrane and hence more cholesterol efflux to apoA-I.

It is also known that apoA-I prefers relatively loosely packed membranes or non-lipid-rafts for its lipidation [36], [37]. We reported previously that ABCA1 is able to generate non-lipid-raft domains [36], perhaps by promoting phospholipid flip-flop [38]. Akt inhibition could introduce further changes in the plasma membrane, thereby promoting cholesterol efflux to apoA-I. We therefore next characterized the plasma membrane microdomain structures.

**Figure 8:**
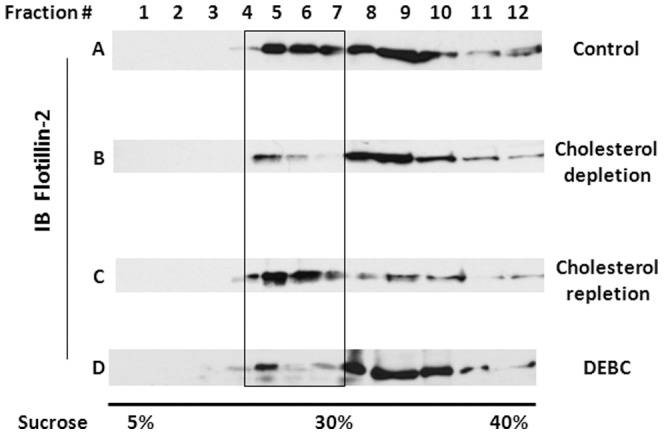
Akt inhibition by DEBC decreases membrane lipid rafts defined by density floatation. ABCA1-expressing BHK cells were untreated (***A***), treated with 5 mM MCD for 30 min to deplete cholesterol (***B***) or treated with 5 mM MCD (30 min) and then 5 mM cholesterol/MCD complex (90 min) (***C***). Some of the cells were also treated with DEBC (50 µM) for 2 h (***D***). Cells were then collected for density floatation as described in the Methods section. The fractions were then analyzed by Western blotting for flotillin-2. Data is representative of more than three independent experiments.

First, membrane domains were separated by detergent-free density floatation. As shown in **Fig. 8** row A, a large portion of flotillin-2, a commonly used lipid raft marker, is in the light density fractions or lipid rafts (fraction 5–7). Upon cholesterol depletion by MCD (5 mM, 30 min), flotillin-2 disappears from these light fractions (row B). Flotillin-2 reappears when cholesterol was replenished by a 90 min incubation with cholesterol/MCD complexs (row C), which is consistent with its being a lipid-raft marker. As mentioned above, DEBC by itself in the absence of apoA-I does not influence cholesterol efflux (no net change of cellular cholesterol contents). However, 2 h DEBC incubation is able to release flotillin-2 from light fractions (row D), similar to cholesterol depleted cells. This suggests a specific disruption of lipid rafts by DEBC.

We have previously demonstrated that one function of ABCA1 is to disrupt lipid rafts without altering cholesterol levels in the plasma membrane [36] and this change of cholesterol packing is necessary for cholesterol efflux to apoA-I [39]. We therefore further tested whether Akt inhibition similarly influenced cholesterol packing in the plasma membrane. Experimentally, this is achieved by cholesterol extraction with MCD for 1 min at 37°C or 30 min at 0°C. 1 min MCD extraction at 37°C is designed to effectively remove plasma membrane cholesterol without significant contribution from intracellular cholesterol pools, thereby assessing plasma membrane cholesterol contents. 30 min MCD extraction at 0°C, on the other hand, preferentially removes cholesterol from loosely packed membranes or non-lipid-raft fractions [36].

**Figure 9:**
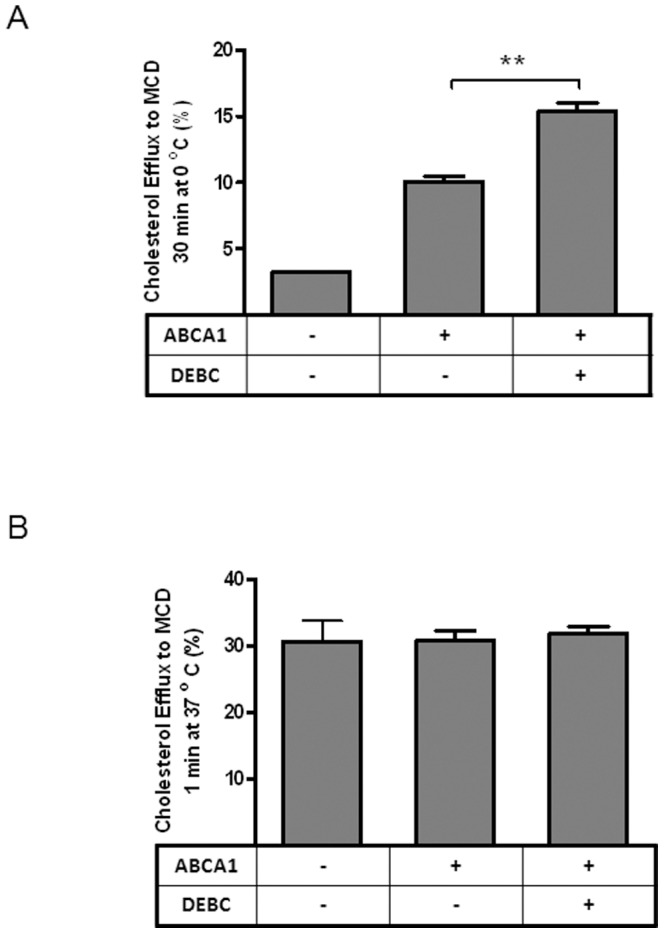
Akt inhibition by DEBC alters cholesterol packing in the plasma membrane. ***A*** *)* BHK-ABCA1 cells were labeled with [^3^H] cholesterol for one day and induced with mifepristone (10 nM) overnight. Cells were then treated with or without DEBC (50 µM) for 2 h before 30 min incubation with MCD at 0°C. Medium was collected and [^3^H] cholesterol in the medium was normalized with total cell associated [^3^H] cholesterol and presented as percentage extraction. ***B*** *)* BHK-ABCA1 cells were similarly treated as in A, except that MCD extraction was performed at 37°C for 1 min. Data presented as the average of triplicate wells with standard deviation and representative of at least three independent experiments.

Consistent with our previous reports, ABCA1 expression increases the amount of cholesterol extracted by MCD at 0°C (**Fig. 9 A**) but, at 37°C, 1 min MCD removes the equal amount of cholesterol, regardless of ABCA1 expression (**Fig. 9 B**). DEBC treatment does not alter ABCA1 expression (Fig. 1 A) or plasma membrane cholesterol levels, judging by 1 min cholesterol extraction at 37°C (**Fig. 9 B** **)**. However, it significantly enhances the amount of cholesterol extracted by MCD at 0°C (**Fig. 9 A**). Together with the density floatation experiment above (Fig. 8), it is plausible that Akt inhibition by DEBC further disrupts lipid rafts in the plasma membrane of ABCA1-expressing cells. This expands the membrane fractions favorable for apoA-I to interact with, which leads to more efficient cholesterol efflux.

## Discussion

In this report, we presented several lines of evidence indicating that Akt negatively regulated ABCA1 function. First, three structurally unrelated Akt inhibitors all significantly increase cholesterol efflux to apoA-I. This effect was strictly ABCA1-dependent, as Akt inhibitors had no effect on either non-ABCA1 expressing cells or cells expressing a defective mutant of ABCA1.

Secondly, the effect of Akt inhibition was applicable to various cell types that express endogenous ABCA1, including macrophages, pancreatic β cells, hepatocytes and MEFs. Cholesterol efflux to apoA-I is known to be essential for maintaining physiological functions in these cell types, as tissue-specific ABCA1 deletion results in foam cell formation (macrophages) [40], diabetes (pancreatic β cells) [41] and extremely low HDL (hepatocytes) [42].

Third, Akt requires mTORC1 to suppress cholesterol efflux to apoA-I. Indeed, the effect of Akt inhibitor was largely muted in TSC2^-/-^ cells, where mTORC1 activity is constitutively elevated and no longer subject to Akt regulation. TSC2^-/-^ cells was also unable to efficiently efflux cholesterol to apoA-I, even though ABCA1 was adequately expressed.

Forth, the connection between Akt and cholesterol efflux was likely autophagy. mTORC1 is one of the major regulators of autophagy. Elevated mTORC1 activity could suppress autophagy and Akt inhibition, on the other hand, could effectively release this suppression. We indeed observed increased LC3-II and decreased CE contents upon Akt inhibition. Interestingly, low doses of LY294002 could block mTORC1 activation (Fig. 4) but failed to effectively activate autophagy (Fig. 7 A). This is consistent with our observation that only high dosages of LY294002 were able to enhance cholesterol efflux (Fig. 2 B). Lastly, Akt inhibition enlarges the non-lipid raft pool of the plasma membrane, which supports increased capacity to efflux cholesterol to apoA-I.

Taken together, we conclude that Akt is a significant regulator of ABCA1 function, such as cholesterol efflux to apoA-I. Akt inhibition most likely accelerates autophagy through suppressing mTORC1, whereby increasing CE hydrolysis and intracellular cholesterol trafficking. Akt inhibition also generates a well-conditioned plasma membrane (less lipid rafts), which enables apoA-I to acquire cholesterol more efficiently.

It should be emphasized that our conclusion is primarily derived from pharmacological studies. As almost always, pharmacological inhibitors could have significant off-target effects, even if they are structurally unrelated. We therefore could not entirely rule out off-target effects by these Akt inhibitors. However, pharmacological inhibitors do have the advantage of acting acutely, such as 2 h used in this study, which offers a unique window of opportunity to delineate the primary effects of target proteins. This is in contrast with long-term inhibition as in genetic ablation or knockdown. For major signaling hubs like Akt, long-term knockout or even knockdown could trigger significant compensatory mechanisms. In this regard, pharmacological inhibitors should be an important and necessary part of research, in addition to genetic manipulations, in order to fully understand the precise sequences of events.

In mammalian cells, Akt is a family of three serine/threonine protein kinases (Akt1, Akt2 and Akt3) that regulate a wide range of cellular functions, including cell survival, proliferation, differentiation and metabolism [43], [44]. These isoforms share the same domain structures and a very high sequence homology. Although the majority of research does not distinguish isoforms, it is increasingly evident that each isoform has significant non-overlapping functions. Most strikingly, individual Akt1, Akt2 or Akt3 knockout mice exhibit distinct phenotypes including metabolic and lipogenesis dysregulation [45]–[47]. We do not currently understand which Akt isoform might be involved in suppressing cholesterol efflux to apoA-I. Isoform-specific antibodies and, particularly, antibodies against phosphorylated Akt isoforms, are either not currently available or of high enough quality for detailed studies in our hands (data not shown). Nevertheless, it is interesting to note that casein kinase 2 (CK2) suppresses cholesterol efflux to apoA-I [13]. CK2 is recently shown to specifically phosphorylate and activate Akt1 [48]. It remains to be seen if CK2 requires Akt1 to suppress ABCA1 function. It is also noteworthy that Akt2 ablation, not Akt1, is reported to promote alternative activation (M2) in macrophages [49]. We recently show that ABCA1 could similarly promote M2 polarization, parallel to its function in cholesterol efflux [50]. It is not clear yet whether ABCA1 directly modulates Akt activities, particularly the activities of each isoform. Akt is mostly studied and understood in the context of cancer. Even there, it is increasingly apparent that each of Akt isoforms plays highly specific and frequently opposing roles. Another important advance in recent years is the distinct roles that each Akt isoform plays in metabolic diseases and atherosclerosis [45]–[47]. Future studies are required to explore whether and how ABCA1 or cholesterol influences the Akt activities in an isoform-specific manner and vice versa.

Interestingly, there is a growing body of evidence that growth factors through PI3K/Akt directly influence lipid metabolism [51]. For example, insulin activates SREBP1-c through mTORC1 [52]. Although most studies have focused on SREBP-1c, insulin signaling was recently shown to be necessary for SREBP-2 activation [53], which is also consistent with earlier observations that Akt directly activates SREBP-2 [54]. Thus, it is not surprising that growth factors through PI3K/Akt may also suppress cholesterol efflux pathways, in parallel with their role in activating cholesterol/phospholipid synthesis.

In summary, the present study implicates the negative involvement of Akt signaling in ABCA1-mediated cholesterol efflux to apoA-I. Given that mTORC1 mediates this negative regulation, our study suggests a significant interaction between cellular cholesterol homeostasis and metabolic regulation.
